# Evaluation of CT-based radiomics signature and nomogram as prognostic markers in patients with laryngeal squamous cell carcinoma

**DOI:** 10.1186/s40644-020-00310-5

**Published:** 2020-04-22

**Authors:** Linyan Chen, Haiyang Wang, Hao Zeng, Yi Zhang, Xuelei Ma

**Affiliations:** 1 Department of Biotherapy, Cancer Center, State Key Laboratory of Biotherapy, West China Hospital, Sichuan University, and Collaborative Innovation Center, No.37, Guoxue Alley, Chengdu, 610041 People’s Republic of China; 2 Department of Otolaryngology, Head and Neck Surgery, West China Hospital, Sichuan University, Chengdu, People’s Republic of China

**Keywords:** Laryngeal cancer, Head and neck cancer, Nomograms, Prognosis

## Abstract

**Background:**

The aim of this study was to evaluate the prognostic value of radiomics signature and nomogram based on contrast-enhanced computed tomography (CT) in patients after surgical resection of laryngeal squamous cell carcinoma (LSCC).

**Methods:**

All patients (*n* = 136) were divided into the training cohort (*n* = 96) and validation cohort (*n* = 40). The LASSO regression method was performed to construct radiomics signature from CT texture features. Then a radiomics nomogram incorporating the radiomics signature and clinicopathologic factors was established to predict overall survival (OS). The validation of nomogram was evaluated by calibration curve, concordance index (C-index) and decision curve.

**Results:**

Based on three selected texture features, the radiomics signature showed high C-indexes of 0.782 (95%CI: 0.656–0.909) and 0.752 (95%CI, 0.614–0.891) in the two cohorts. The radiomics nomogram had significantly better discrimination capability than cancer staging in the training cohort (C-index, 0.817 vs. 0.682; *P* = 0.009) and validation cohort (C-index, 0.913 vs. 0.699; *P* = 0.019), as well as a good agreement between predicted and actual survival in calibration curves. Decision curve analysis also suggested improved clinical utility of radiomics nomogram.

**Conclusions:**

Radiomics signature and nomogram showed favorable prediction accuracy for OS, which might facilitate the individualized risk stratification and clinical decision-making in LSCC patients.

## Background

Laryngeal cancer is a common malignant tumor of head and neck, with an incidence of 26,300 new cases and 14,500 deaths in China [1]. And laryngeal squamous cell carcinoma (LSCC) accounts for approximately 85–95% of laryngeal cancer cases [2]. The five-year survival rate of laryngeal cancer was about 65% [3]. However, the survival rate did not increase significantly despite the improvement of early diagnosis and therapy [4, 5]. The pathological tumor node metastasis (TNM) staging is most widely used prognostic factor for long-term survival [6]. However, pathological evaluation might fail to predict the response to non-surgical treatments. Therefore, the novel and effective prognostic markers are of great importance for improving risk stratification and optimizing therapeutic strategies in LSCC patients.

It is well known that the development, therapeutic response and prognosis of tumors are associated with the intratumoral heterogeneity, such as gene mutation-expression, cellular histology, angiogenesis and tumor microenvironment [7]. Recent studies have focused on the texture analysis of computed tomography (CT), which showed the potential prognostic value in several tumors, including lung cancer and liver cancer [8, 9]. Image texture is a set of metrics calculated by numerous mathematical calculations, which provides information about the spatial arrangement and variation of pixel intensities in gray-scale images [10]. Moreover, the radiomics analyses are performed on image processing systems to estimate the texture features in CT images of tumors, and represent the intratumoral heterogeneity [11, 12]. The nomogram, also known as alignment diagram, which uses several scale lines to represent multiple predictors, then expresses the interrelation between variables and calculates the probability of events based on multivariate regression model. In addition, published studies have suggested that radiomics nomogram had significant predictive accuracy for lymph node metastasis and survival outcomes in cancer patients [13, 14].

Although the individual CT texture parameters have showed significant predictive role of survival and treatment failure in head and neck squamous cell carcinoma (HNSCC) [15, 16]. The application of radiomics signature combined with multiple CT texture markers in LSCC patients has not been well discussed. Therefore, we aimed to build and validate whether the radiomics signature and relevant nomogram could be used as effective prognostic markers for overall survival (OS) in LSCC patients.

## Materials and methods

### Patients

The medical records of LSCC patients between January 2011 to December 2015 were reviewed. All patients underwent surgical resection and pathological examination. The cancer staging was confirmed based on 8th edition AJCC-TNM stage [17]. We included patients who had preoperative contrast-enhanced CT. However, the CT images of non-identifiable tumors or image artifacts were excluded. Patients who underwent neoadjuvant chemoradiotherapy or being lost to follow-up were also excluded. Based on the random number, patients were divided into the training set and the validation set at a proportion of 7:3. The patients’ demographics (age and gender), tumor characteristics (location, stage and histological grade) and treatments were compared between two cohorts. All the patients were followed up until death or last follow-up of December 2017. We analyzed OS as the endpoint, which meant the period from definite diagnosis to death or last follow-up.

### CT imaging protocols and texture analysis

Contrast-enhanced CT images were obtained via a Philips Brilliance 16-slice CT scanner (Philips Medical System, US). All patients received intravenously nonionic contrast agent (1.5–2.0 ml/kg, iohexol; Beijing Beilu Pharmaceutical, China). This study used a free Java software called LIFEx (Orsay, France, http://www.lifexsoft.org) to extract radiomics features from multiple and consecutive CT images with 1 mm slice thickness [18]. The viewer of LIFEx supports the synchronized display of 3 directional slices (coronal, sagittal and transaxial) and maximum display of intensity projection. Images were labeled with random number and reviewed by blinded method. An independent experienced radiologists (HW) manually drew a region of interest (ROI) around the tumor border. The cervical lymph nodes were not involved. Finally, 36 texture features were extracted from LifeX, as described below [11].

(1) First order metrics: histogram and geometry-based features: skewness (degree of asymmetry of gray-level distribution), kurtosis (peakedness of distribution), entropy (disorder or randomness of pixel distribution), energy (homogeneity or uniformity of pixel distribution), sphericity (regularity of volume shape) and compacity (compactness of volume shape). (2) Second order metrics: gray-level co-occurrence matrix (GLCM): homogeneity (closeness of voxel pairs), entropy, energy, contrast (local variations), correlation (gray-level linear dependence) and dissimilarity (variation of voxel pairs). (3) Second order metrics: neighborhood gray-level dependence matrix (NGLDM): contrast (spatial rate change of intensity) and coarseness (difference of intensity between regions). (4) Third order metrics: gray-level run length matrix (GLRLM) and gray-level zone length matrix (GLZLM), which were calculated by a single co-occurrence matrix, then provide information about the size of homogenous runs for each gray-level directly in three dimensions.

### Radiomics signature construction and validation

**Fig. 1:**
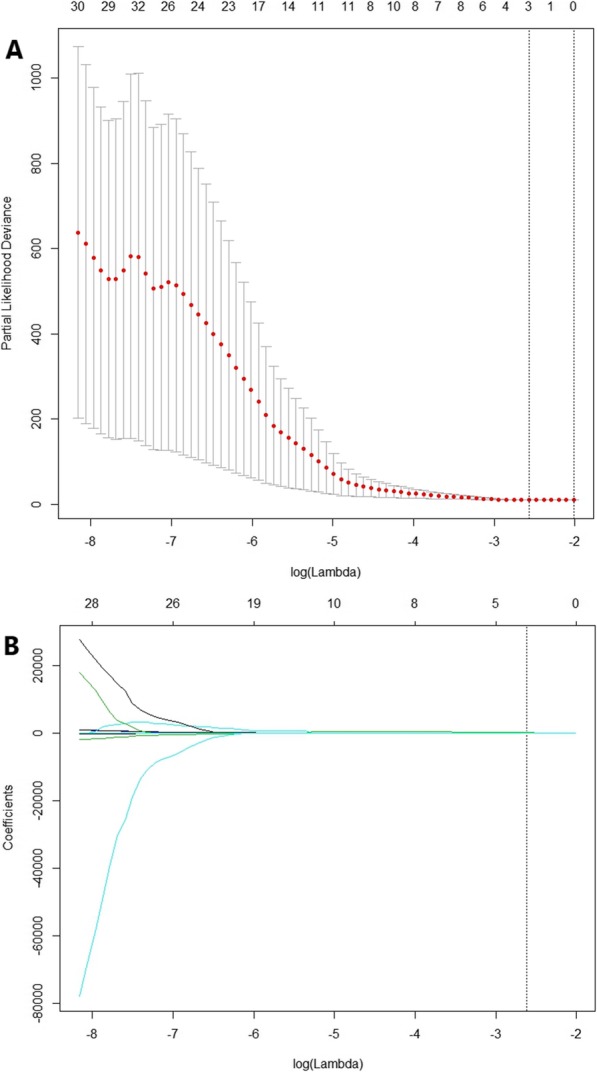
Texture feature selection using LASSO Cox regression. **a** Selection of tuning parameter (λ) in the LASSO model using 10-foldncross-validation with minimum criteria. Partial likelihood deviance was plotted versus log (λ). Vertical line of the optimal values (log (λ) = − 2.573) were drawn based on the minimum criteria and the 1-standard error of the minimum criteria. **b** LASSO coefficient profiles of 36 texture features. Vertical line was plotted at the selected value via 10-fold cross-validation, where optimal λ resulted in 3 nonzero coefficients

The least absolute shrinkage and selection operator (LASSO)-Cox regression algorithm was used to reduce the dimension of high-dimensional data in training dataset [19, 20]. Then we calculated the radiomics signature (Rad-score) by linear combination of features weighted by LASSO coefficients. Three features with nonzero coefficients were selected (Fig. 1). There were high gray-level run emphasis (HGRE), long-run high gray-level emphasis (LRHGE) and zone length non-uniformity (ZLNU). The radiomics score was calculated according to the following formula: 0.000318616 × GLRLM_HGRE+(1.83E-05) × GLRLM_LRHGE + 0.001307454 × GLZLM_ZLNU. The cut-off values of radiomics score were estimated by the area under the curve (AUC) of receiver operating curve (ROC). The Kaplan-Meier survival analysis evaluated the unadjusted association between Rad-score and survival outcome. Then we calculated the hazard ratio (HR) and related 95% confidence interval (CI) by univariate Cox regression analysis for each variable.

### Radiomics nomogram building and assessment

Only significant variables in univariate Cox analyses were further included in nomogram for training cohort. The radiomics nomograms containing radiomics signature and clinicopathologic risk factors were conducted on multivariate Cox regression model. The Harrell’s concordance index (C-index) represented the consistence probability between the observed and predicted survival outcome, which was calculated by a bootstrap validation with 1000 re-samples. The C-index above 0.9 represents high accuracy, the value within 0.7 to 0.9 indicates moderate accuracy, and C-index of 0.5 suggests no predictive ability [21]. In addition, we applied calibration curves and Hosmer-Lemeshow tests to evaluate the goodness-of-fit [22]. These analyses were performed in both training cohort and validation cohort. Finally, the decision curve analysis (DCA) was conducted to measure the net benefits on threshold probabilities in validation group [23]. All above analyses were performed on R version 3.5.3, and *p* value< 0.05 was regarded as statistically significant.

## Results

### Patient characteristics

There were 136 eligible patients (128 male and 8 female) in this study. The median age at the time of initial diagnosis was 60 years (range 30–86 years). There was no distant metastases (M0) in all patients at the first diagnosis. Adjuvant intensity-modulated radiotherapy (IMRT) and cisplatin chemotherapy were used. The median period of follow-up was 42 months (range 4–86 months). There were 20 patients (14.7%) died and 46 patients (33.8%) developed cancer progression. Twenty-seven, seven and four patients suffered local relapse, metastases of cervical lymph nodes and distant organs. The complete characteristics of the training cohort and validation cohort were described in Table 1. There was no significant statistical difference between two cohorts (all *P* > 0.05).

**Table 1:** Characteristics of patients in the training and validation cohorts

Characteristic	Training cohort (***n*** = 96)	Validation cohort (***n*** = 40)	***P*** value
Age			0.925
Median	60 (30–83)	60 (42–86)	
< 60	46 (47.0)	18 (45.0)	
≥ 60	50 (52.1)	22 (55.0)	
Gender			0.906
Male	90 (93.8)	38 (95.0)	
Female	6 (6.25)	2 (5.0)	
Tumor location			0.908
Supraglottic	22 (22.9)	8 (20.0)	
Glottic	71 (74.0)	31 (77.5)	
Subglottic	3 (3.1)	1 (2.5)	
T classification			0.733
T1	25 (26.0)	13 (32.5)	
T2	32 (33.3)	13 (32.5)	
T3	27 (28.1)	8 (20.0)	
T4	12 (12.5)	6 (15.0)	
N classification			0.525
N0	78 (81.3)	35 (87.5)	
N1	11 (11.5)	4 (10.0)	
N2	7 (7.3)	1 (2.5)	
Cancer stage			0.752
I	25 (26.0)	13 (32.5)	
II	28 (29.2)	11 (27.5)	
III	29 (30.2)	9 (22.5)	
IV	14 (14.6)	7 (17.5)	
Histological grade			0.508
Well	37 (38.5)	17 (42.5)	
Moderate	36 (37.5)	17 (42.5)	
Poor	23 (24.0)	6 (15.0)	
Laryngectomy			0.196
Partial	77 (80.2)	28 (29.2)	
Total	19 (19.8)	12 (12.5)	
Adjuvant therapy			0.792
No	67 (69.8)	27 (67.5)	
Yes	29 (30.2)	13 (13.5)	
Follow-up time (mo)			0.959
Median	42 (4–86)	40 (5–82)	
Rad-score			0.646
Median	4.34 (3.74–6.12)	4.29 (3.95–5.98)	

### Assessment of radiomics signature

**Fig. 2:**
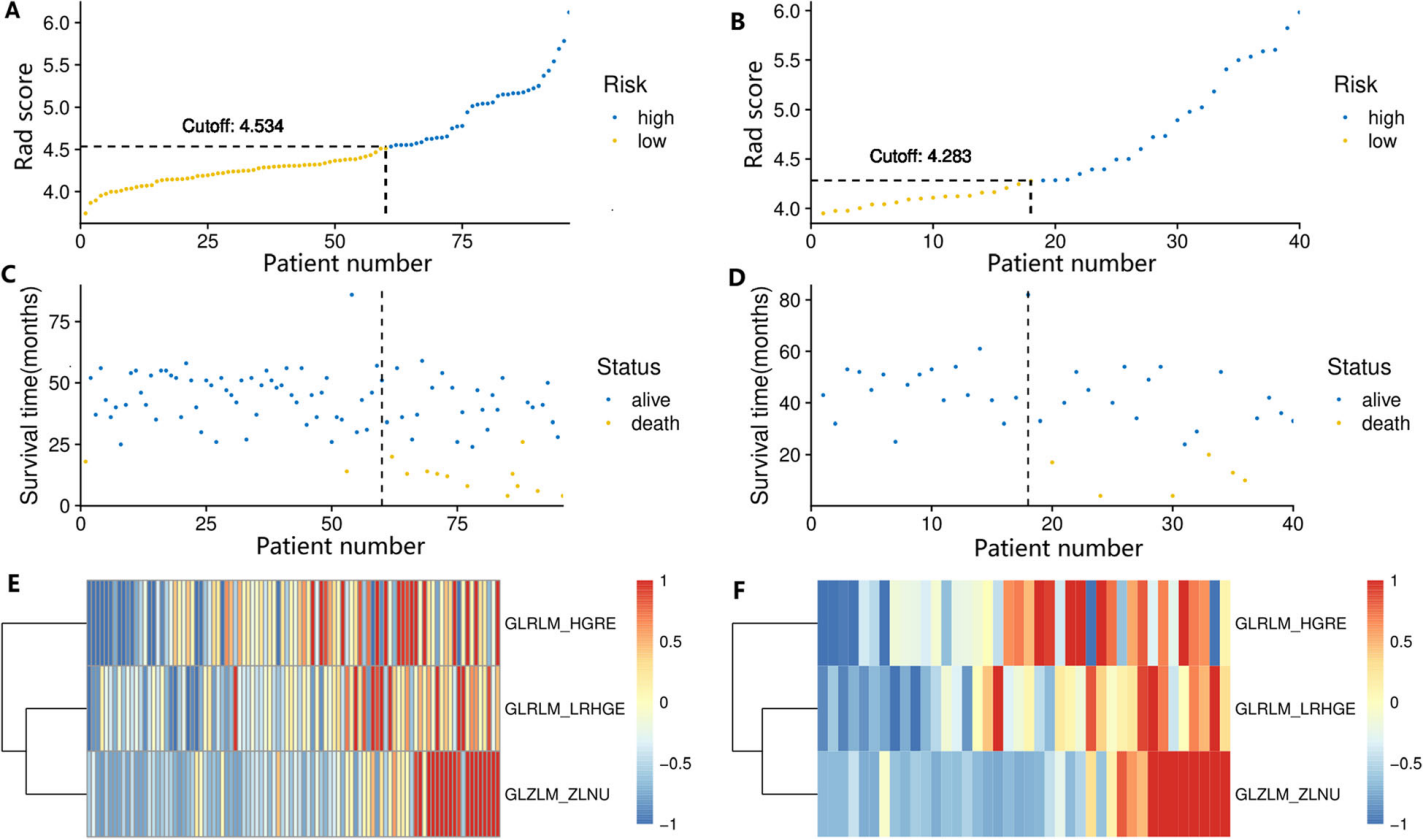
Development of the prognostic index based on radiomics score (Rad-score). Distribution of prognostic index in training cohort (**a**) and validation cohort (**b**). Patients were sorted in numerical order according to Rad-score on the x-axes, and divided into high-risk and low-risk groups. Survival status in training cohort (**c**) and validation cohort (**d**) showed higher proportion of death patients in high-risk group. Heatmap of texture features values in training cohort (**e**) and validation cohort (**f**) tended to be higher in high-risk patients

**Fig. 3:**
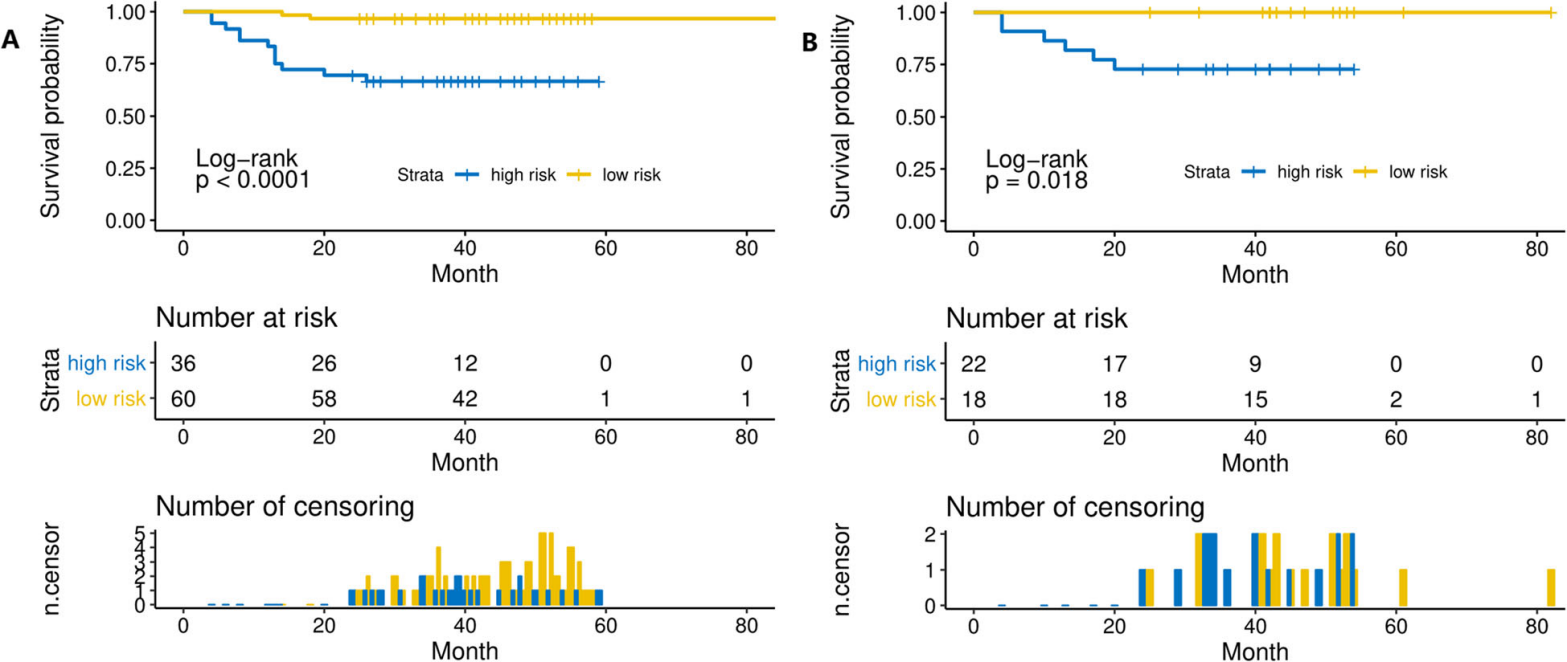
Kaplan-Meier curves with number of risk and censoring for OS in the training cohort (**a**) and validation cohort (**b**). Elevated radiomics scores were significantly associated with poorer OS

Through the LASSO-Cox analysis, three features with nonzero coefficients were selected to calculate the radiomics signature (Rad-score). No significant difference of Rad-score was found between the training cohort and validation cohort (*P* = 0.646). The AUCs were 0.783 (95%CI: 0.646–0.921, *P* = 0.001) for the training cohort and 0.770 (95%CI: 0.617–0.922, *P* = 0.037) for the validation cohort. The optimal cut-off points of Rad-score were 4.534 in training cases and 4.283 in validation cases, then we classified patients into high-risk or low-risk groups (Fig. 2a,b). It showed that high-risk groups had more patients in death status (Fig. 2c,d). And the heatmaps demonstrated that three texture features generally tended to be higher value in high-risk patients (Fig. 2e,f). The elevated radiomics signature was significantly related with worse OS in the training cohort (HR = 11.98, 95%CI: 2.68–53.56, *p* = 0.001; Fig. 3a) and the validation cohort (HR = 6.75, 95%CI: 1.35–33.70, *p* = 0.020; Fig. 3b).

### Validation of radiomics nomogram

**Fig. 4:**
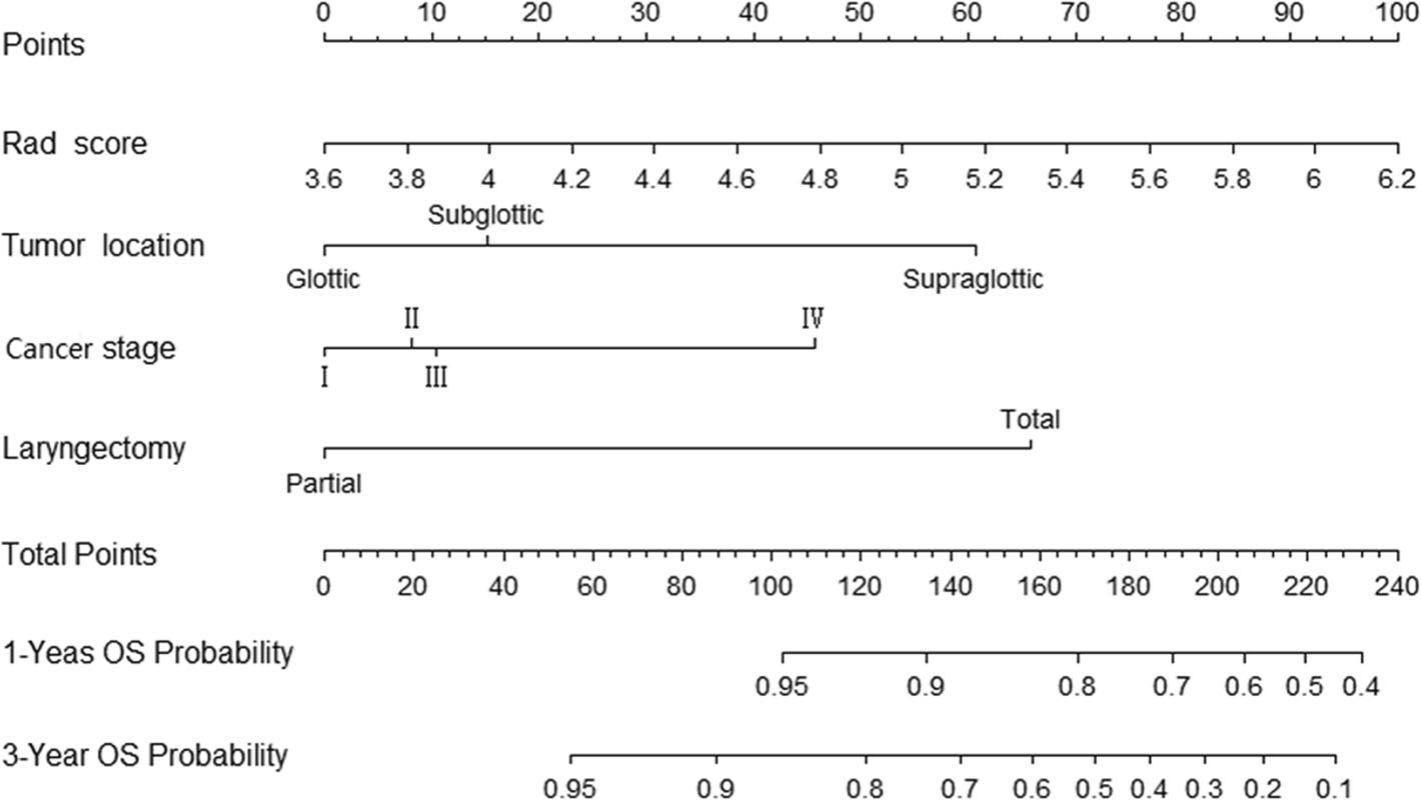
Radiomics nomogram for the prediction of 1-year and 3-year OS based on the training cohort

**Fig. 5:**
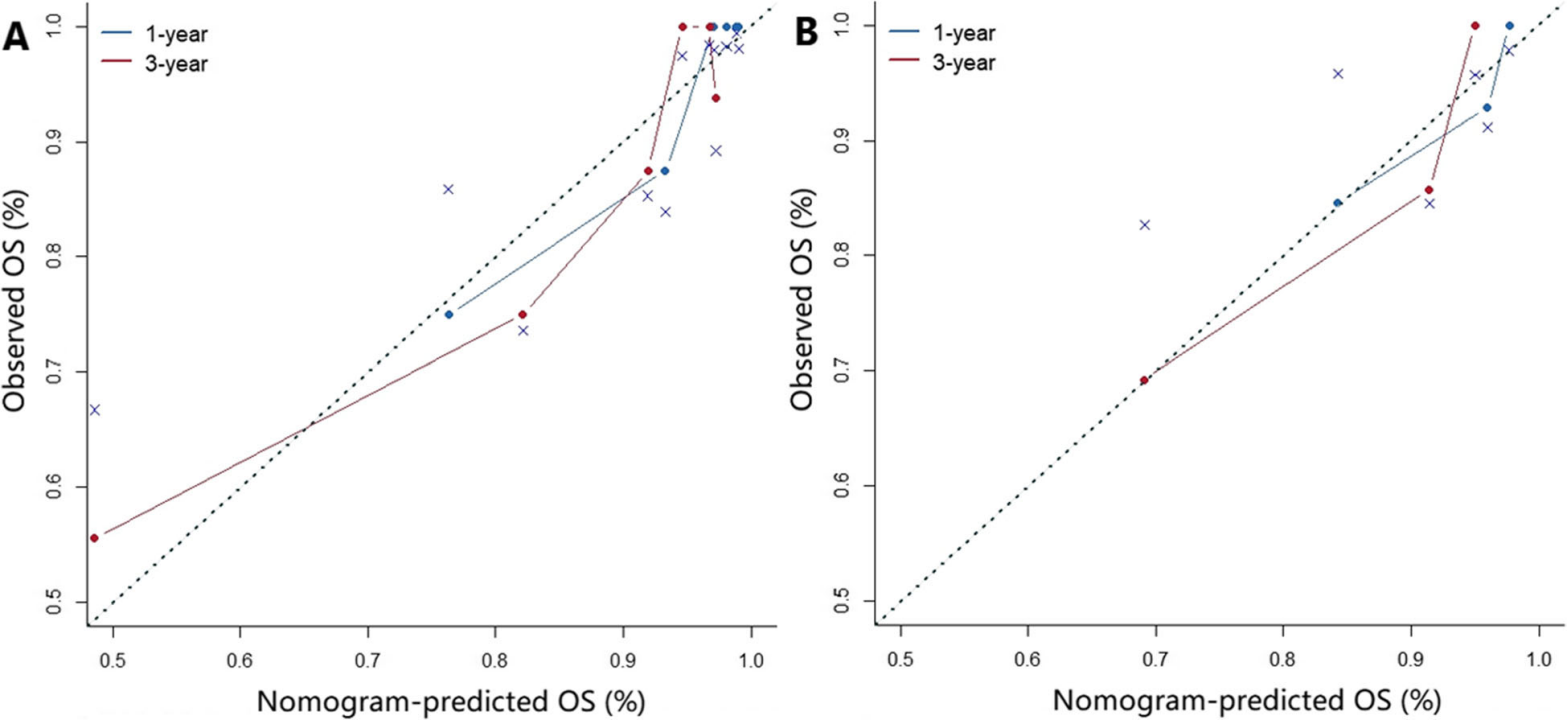
Calibration curves of radiomics nomogram in the training cohort (**a**) and validation cohort (**b**). The diagonal dotted line represented a perfect prediction by an ideal model, and solid line represented performance of the nomogram. The closer fit between the diagonal dotted lines and solid lines showed good prediction of 1-year and 3-year OS

**Fig. 6:**
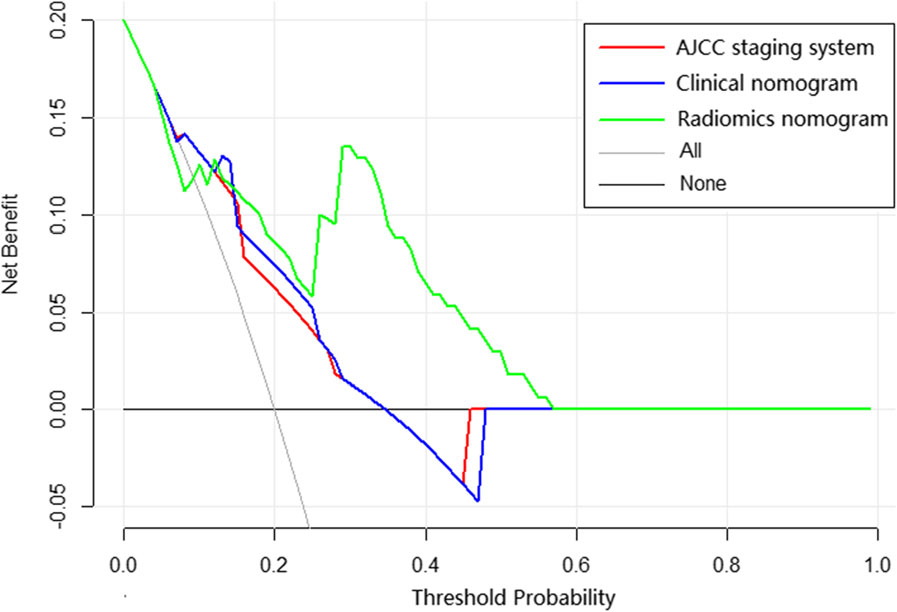
Decision curve analysis for each model based on the validation dataset. The risk probability of death was recorded as Pi. When Pi reached a certain threshold probability (Pt), it was defined as positive, and some intervention were taken. The net benefit was calculated by summing the benefits of intervention in true positive proportion (**a**) and loss benefit of unnecessary treatment in false positive proportion (**b**). Net benefit = a-b [Pt/(1-Pt)]. The horizontal black line represented all negative samples and no intervention. The gray dotted oblique represented intervention of all patients. The radiomics nomogram had the highest net benefit compared with all-treat scheme or non-treat scheme and other models across the range of 15–55% in Pt. For example, if the Pt reached 30%, the net benefit was about 0.10 when using the radiomics nomogram to determine whether to perform therapies

The clinical nomogram contained significant characteristics of tumor location, TN stage and laryngectomy types in the training set (Table 2). The radiomics signature and above characteristics were further included in the radiomics nomogram (Fig. 4). Then we compared the C-indexes of different models with tumor staging as the reference (Table 3). The radiomics nomogram model had higher accuracy compared with cancer staging in the training cohort (*P* = 0.009), which also revealed good predictive performance than cancer staging (*P* = 0.019) and clinical nomogram (*P* = 0.008) in the validation cohort. In training set, the calibration curves of 1-year and 3-year OS and nonsignificant Hosmer-Lemeshow test (*P* = 0.833; *P* = 0.706; Fig. 5a) showed good agreement between predicted and actual OS. Moreover, the radiomics nomogram performed well in the validation set (*P* = 0.952; *P* = 0.091; Fig. 5b). The DCA illustrated that when the threshold probability was approximately within 15 to 55%, the radiomics nomogram had a better net benefit for decision-making than other models (Fig. 6).

**Table 2:** Univariate Cox analysis of clinicopathological characteristics

Parameters	Cut-off value	Training cohort			Validation cohort		
		HR	95% CI	***P*** value	HR	95% CI	***P*** value
Age (year)	< 60 vs. ≥ 60	2.40	0.75–7.65	0.139	1.03	0.95–1.10	0.537
Gender	Female vs. Male	0.30	0.04–2.27	0.242	0.46	0.08–4.06	0.565
Tumor location	Glottic vs. Non-glottic	5.86	1.96–17.51	0.002	1.67	0.31–9.14	0.553
T classification	T1-T2 vs. T3-T4	2.06	0.72–5.96	0.178	1.90	0.38–9.41	0.432
N classification	N0 vs. N1-N2	2.64	0.88–7.88	0.082	4.50	0.82–14.76	0.084
Cancer stage	I-II vs. III-IV	2.56	1.03–6.49	0.042	3.21	0.76–7.53	0.178
Histological grade	Well-moderate vs. Poor	2.56	0.88–7.38	0.092	1.05	0.49–3.58	0.692
Laryngectomy	Partial vs. Total	7.34	2.43–20.34	< 0.001	2.38	1.02–8.25	0.048
Adjuvant therapy	No vs. Yes	0.91	0.29–2.90	0.871	2.29	0.46–11.35	0.311

**Table 3:** Discrimination performance of models

Model	Training cohort			Validation cohort		
	C-index	95% CI	***P*** value	C-index	95% CI	***P*** value
Radiomics signature	0.782	0.656–0.909	0.170	0.752	0.614–0.891	0.456
Clinical nomogram	0.802	0.690–0.914	0.007	0.807	0.630–0.985	0.192
Radiomics nomogram	0.817	0.693–0.942	0.009	0.913	0.833–0.992	0.019
AJCC staging system	0.682	0.553–0.812	Reference	0.699	0.458–0.941	Reference

## Discussion

In present study, we extracted texture features of preoperative contrast-enhanced CT images, and used machine-learning method to obtain a 3 features-based radiomics signature. This study extended the individual texture analysis to the survival assessment of radiomics based on multiple texture features. The results showed that radiomics signature was a potential prognostic marker for OS. Moreover, the radiomics nomogram incorporating radiomics signature and other clinicopathological characteristics was practical in survival prediction, which had improved discrimination ability than traditional cancer staging in both training cohort and validation cohort. Therefore, it indicated that radiomics signature and nomogram had additional prognostic value for OS in patient with LSCC.

Computer tomography (CT) is a widely applied instrument for noninvasive diagnosis and staging of laryngeal cancer before treatment. Plain CT images reflect the non-homogeneity of intratumoral tissue and cell density due to necrosis, hemorrhage and cystic degeneration [24, 25]. Additionally, enhanced scans reflect the heterogeneity of vascular supply, with increased blood supply in some areas and decreased blood supply in others [26, 27]. Based on CT texture analysis, intratumoral heterogeneity can be translated into the heterogeneity in spatial distribution of density pixels, which was related with pathological grade, tumor aggressiveness, tumoral biological index (e.g. hypoxia markers, VEGF) as well as prognosis and therapeutic response [28–30]. For head and neck cancer, most of previous studies separately evaluated the prognostic value of individual CT texture parameters. For example, the entropy and skewness were independently associated with OS in HNSCC patients undergoing TPF chemotherapy [15]. In histogram analysis, a pixel distribution with higher kurtosis, energy and entropy, and a positive or negative skewness indicated the enhancement of tumors heterogeneity [31, 32]. The high homogeneity of PET-CT images also was revealed as predictors of progression-free survival in pharynx cancer [33]. In terms of third order metrics, significant differences of GLRLM features were found between regional control group and local recurrence group in HNSCC patients treated with chemoradiotherapy [16].

As mentioned, there were hundreds and thousands of texture features, and their prognostic roles were widely different in previous studies. Therefore, the analyses of single texture features were time-consuming and inefficient. Furthermore, considering the potential overfit-ting of radiomics features, it was meaningful to reduce and shrink features before model building. The integrative radiomics signature would facilitate the application of CT texture feature. In our study, the radiomics signature demonstrated favorable discriminative ability in the training cohort (AUC = 0.783, C-index = 0.782) and validation cohort (AUC = 0.770, C-index = 0.752) compared with previous studies (AUC = 0.66–0.69) [34]. In addition, an MRI-based radiomics study of HNSCC concluded moderate C-indexes of 0.73 in training cohort and 0.71 in validation cohort [35]. These results suggested that radiomics heterogeneity of primary mass observed in CT and MRI images might be helpful to judge prognosis and guide treatment in cancer patients. No final conclusion has yet been reached, which still needs more studies to determine whether radiomics signature could be effective predictor of prognosis or not.

The TNM staging was traditionally suggested as an independent prognostic predictor in HNSCC patients [36]. Primary site of LSCC was also associated with survival results. The prognosis of supraglottic laryngeal cancer was poorer than that of subglottic and supraglottic cancer, which possibly due to common cervical lymph node metastasis in supraglottic laryngeal cancer [37]. In our study, survival results of patients undergoing partial laryngectomy was better than patients undergoing total laryngectomy. It was probably because patients with early-stage cancer often received partial resection than total resection. Then we incorporated the radiomics signature and above clinical factors in nomogram to improve the survival prediction. It has been suggested that single risk factor without model may be difficult to comprehensively evaluate the postoperative outcome of different patients, thus a prognostic model is necessary to consider multiple risk factors for each patient, such as nomogram [14]. Previous MRI-based radiomics studies also showed significantly higher C-indexes of radiomics nomogram than TNM staging [34, 38]. However, the effectiveness of radiomics nomogram still requires further investigation.

There were several shortcomings in the present study. Firstly, due to retrospective design and small sample of single-center, the potential selection bias cannot be excluded, which limited the accuracy and reliability of results. Secondly, the variation between observed images should be considered when drawing the outline of ROI areas. The computer-aided software of this study may help to reduce variation to some degree. Thirdly, although there was no significant difference in characteristics between the two cohorts, the variables including therapeutic strategies and complications might act as potential confounders. Furthermore, there were many kinds of texture features and images processing software, thus unifying the texture analysis would help to achieve robust results and spread the application.

## Conclusions

Contrast-enhanced CT radiomics signature was independently associated with overall survival in LSCC patients. The radiomics nomogram might act as a noninvasive and effective model to improve the individualized prognostic evaluation and treatment strategies. Therefore, more researches are warranted for better estimation, especially the large-scale prospective and multi-center studies.

## Data Availability

The datasets during and/or analysed during the current study available from the corresponding author on reasonable request.

## Abbreviations

LSCC: Laryngeal squamous cell carcinoma
CT: computed tomography
HNSCC: head and neck squamous cell carcinoma
OS: overall survival
ROI: region of interest
GLCM: gray-level co-occurrence matrix
NGLDM: Second order metrics: neighborhood gray-level dependence matrix
GLRLM: gray-level run length matrix
GLZLM: gray-level zone length matrix
LASSO: least absolute shrinkage and selection operator
AUC: area under the curve
ROC: receiver operating curve
HR: hazard ratio
CI: confidence interval
C-index: concordance index
DCA: decision curve analysis
HGRE: High gray-level run emphasis
LRHGE: long-run high gray-level emphasis
ZLNU: zone length non-uniformity

## References

[1] Chen W, Zheng R, Baade PD (2016). Cancer statistics in China, 2015. CA Cancer J Clin.

[2] Schorn VJ, Miles BA (2014). Laryngeal Squamous Cell Carcinoma.

[3] Muller P, Belot A, Morris M, Rachet B, Cancer Research UK Cancer survival group, London School of Hygiene and Tropical Medicine. Net survival and the probability of cancer death from rare cancers Available from http://csg.lshtm.ac.uk/rare-cancers/. Accessed 20 Sept 2016.

[4] Siegel R, Ma J, Zou Z, Jemal A (2014). Cancer statistics, 2014. CA Cancer J Clin.

[5] Hoffman HT, Porter K, Karnell LH (2006). Laryngeal cancer in the United States: changes in demographics, patterns of care, and survival. Laryngoscope.

[6] Balch CM, Soong SJ, Gershenwald JE (2001). Prognostic factors analysis of 17,600 melanoma patients: validation of the American joint committee on Cancer melanoma staging system. J Clin Oncol.

[7] Michor F, Polyak K (2010). The origins and implications of intratumor heterogeneity. Cancer Prev Res (Phila).

[8] Ahn SY, Park CM, Park SJ (2015). Prognostic value of computed tomography texture features in non-small cell lung cancers treated with definitive concomitant chemoradiotherapy. Investig Radiol.

[9] Cozzi L, Dinapoli N, Fogliata A (2017). Radiomics based analysis to predict local control and survival in hepatocellular carcinoma patients treated with volumetric modulated arc therapy. BMC Cancer.

[10] Tang X (1998). Texture information in run-length matrices. IEEE Trans Image Process.

[11] Nardone V, Tini P, Nioche C (2018). Texture analysis as a predictor of radiation-induced xerostomia in head and neck patients undergoing IMRT. Radiol Med.

[12] Buvat I, Orlhac F, Soussan M (2015). Tumor texture analysis in PET: where do we stand?. J Nucl Med.

[13] Huang YQ, Liang CH, He L (2016). Development and validation of a Radiomics Nomogram for preoperative prediction of lymph node metastasis in colorectal Cancer. J Clin Oncol.

[14] Huang Y, Liu Z, He L (2016). Radiomics signature: a potential biomarker for the prediction of disease-free survival in early-stage (I or II) non-small cell lung Cancer. Radiology..

[15] Zhang H, Graham CM, Elci O (2013). Locally advanced squamous cell carcinoma of the head and neck: CT texture and histogram analysis allow independent prediction of overall survival in patients treated with induction chemotherapy. Radiology..

[16] Kuno H, Qureshi MM, Chapman MN (2017). CT texture analysis potentially predicts local failure in head and neck squamous cell carcinoma treated with Chemoradiotherapy. AJNR Am J Neuroradiol.

[17] Lydiatt WM, Patel SG, O'Sullivan B (2017). Head and neck cancers-major changes in the American joint committee on cancer eighth edition cancer staging manual. CA Cancer J Clin.

[18] Nioche C, Orlhac F, Boughdad S (2018). LIFEx: a freeware for Radiomic feature calculation in multimodality imaging to accelerate advances in the characterization of tumor heterogeneity. Cancer Res.

[19] Sauerbrei W, Royston P, Binder H (2007). Selection of important variables and determination of functional form for continuous predictors in multivariable model building. Stat Med.

[20] Tibshirani R (1997). The lasso method for variable selection in the cox model. StatMed..

[21] Pencina MJ, D'Agostino RB (2004). Overall C as a measure of discrimination in survival analysis: model specific population value and confidence interval estimation. Stat Med.

[22] Kramer AA, Zimmerman JE (2007). Assessing the calibration of mortality benchmarks in critical care: the Hosmer-Lemeshow test revisited. Crit Care Med.

[23] Vickers AJ, Cronin AM, Elkin EB (2008). Extensions to decision curve analysis, a novel method for evaluating diagnostic tests, prediction models and molecular markers. BMC Med Inform Decis Mak.

[24] Ganeshan B, Goh V, Mandeville HC (2013). Non-small cell lung Cancer: Histopathologic correlates for texture parameters at CT. Radiology..

[25] Sun J, Yu XR, Shi BB, Zheng J, Wu JT. CT features of retroperitoneal solitary fibrous tumor: report of three cases and review of the literature. World J Surg Oncol. 2014;12:324. 10.1186/1477-7819-12-324 PMC4282173 25351104

[26] Nordsmark M, Overgaard M, Overgaard J (1996). Pretreatment oxygenation predicts radiation response in advanced squamous cell carcinoma of the head and neck. Radiother Oncol.

[27] Nelson DA, Tan TT, Rabson AB, Anderson D, Degenhardt K, White E (2004). Hypoxia and defective apoptosis drive genomic instability and tumorigenesis. Genes Dev.

[28] Skogen K, Ganeshan B, Good C, Critchley G, Miles K (2013). Measurements of heterogeneity in gliomas on computed tomography relationship to tumour grade. J Neuro-Oncol.

[29] Swinson DE, O'Byrne KJ (2006). Interactions between hypoxia and epidermal growth factor receptor in non-small-cell lung cancer. Clin Lung Cancer.

[30] Goh V, Sanghera B, Wellsted DM, Sundin J, Halligan S (2009). Assessment of the spatial pattern of colorectal tumour perfusion estimated at perfusion CT using two-dimensional fractal analysis. Eur Radiol.

[31] Yun G, Kim YH, Lee YJ, Kim B, Hwang JH, Choi DJ (2018). Tumor heterogeneity of pancreas head cancer assessed by CT texture analysis: association with survival outcomes after curative resection. Sci Rep.

[32] Ganeshan B, Skogen K, Pressney I, Coutroubis D, Miles K (2012). Tumour heterogeneity in oesophageal cancer assessed by CT texture analysis: preliminary evidence of an association with tumour metabolism, stage, and survival. Clin Radiol.

[33] Fujima N, Hirata K, Shiga T (2018). Integrating quantitative morphological and intratumoural textural characteristics in FDG-PET for the prediction of prognosis in pharynx squamous cell carcinoma patients. Clin Radiol..

[34] Parmar C, Grossmann P, Rietveld D, Rietbergen MM, Lambin P, Aerts HJ (2015). Radiomic machine-learning classifiers for prognostic biomarkers of head and neck Cancer. Front Oncol.

[35] Yuan Y, Ren J, Shi Y, Tao X. MRI-based radiomic signature as predictive marker for patients with head and neck squamous cell carcinoma. Eur J Radiol. 2019;117:193–98. 10.1016/j.ejrad.2019.06.019 31307647

[36] Raitiola H, Pukander J, Laippala P (1999). Glottic and supraglottic laryngeal carcinoma: differences in epidemiology, clinical characteristics and prognosis. Acta Otolaryngol.

[37] Jin T, Hu WH, Guo LB (2011). Treatment results and prognostic factors of patients undergoing postoperative radiotherapy for laryngeal squamous cell carcinoma. Chin J Cancer.

[38] Zhang B, Tian J, Dong D, Gu D, Dong Y, Zhang L (2017). Radiomics features of multiparametric MRI as novel prognostic factors in advanced nasopharyngeal carcinoma. Clin Cancer Res.

